# Therapeutic potential of Chinese medicinal herbs stimulating osteogenic differentiation of bone marrow-derived mesenchymal stem cells in osteoporosis 

**DOI:** 10.3389/fphar.2024.1423555

**Published:** 2024-07-31

**Authors:** Hui Wang, Kai Shan, Yan Li, Sinuo Wu, Chunman Zhou, Shan Tao, Meijuan Wang, Xiaochun Kang, Liang Zhou, Zhongxi Lyu, Ningcen Li

**Affiliations:** ^1^ Xi’an Hospital of Traditional Chinese Medicine, Xi’an, Shanxi, China; ^2^ Department of Traditional Chinese Medicine, The First Clinical Medical College of Shaanxi University of Chinese Medicine, Xianyang, Shanxi, China; ^3^ Qingdao Hospital of Traditional Chinese Medicine (Qingdao Hiser Hospital), Qingdao, Shandong, China; ^4^ Research Center of Experimental Acupuncture Science, Tianjin University of Traditional Chinese Medicine, Tianjin, China; ^5^ Acupuncture and Moxibustion Department, Nanchang Hongdu Hospital of Traditional Chinese Medicine, Nanchang, Jiangxi, China

**Keywords:** traditional Chinese medicine, Chinese medicinal herbs, osteoporosis, bone marrow-derived mesenchymal stem cells, synergistic efficiency enhancement

## Abstract

Osteoporosis (OP) is a common and complex chronic metabolic disease with an increasing incidence rate, which has markedly increased the human health burden worldwide. The predominant cause of OP is an imbalance between osteoblasts (OB) and osteoclasts (OC). Studies on the correlation between bone marrow-derived mesenchymal stem cells (BMSCs) and OP have indicated that BMSCs-induced OB differentiation is an important pathway for bone tissue renewal. Chinese medicinal herbs have been used for centuries to treat various types of OPs because they are safer and more effective. The *in vivo* and *in vitro* experiments have confirmed that these herbs or their primary phytochemicals may exert therapeutic effects by stimulating BMSCs differentiation, which restores OB and OP balance, inhibits adipocyte differentiation, exerts anti-inflammatory and antioxidant effects, regulates the immune system, etc. This review summarizes the research on how Chinese medicinal herbs or their primary phytochemicals treat OP by stimulating BMSC differentiation and provides a scientifically reliable basis and perspective for their future clinical application.

## 1 Introduction

Osteoporosis (OP) is a common and complex chronic metabolic disease, which frequently affects middle-aged and elderly people. Furthermore, with an increasing aging population, the incidence rate of OP is also increasing (Khandelwal and Lane, 2023). Moreover, it is a bone metabolic disease with different etiologies, it develops slowly in years and is characterized by bone loss and brittle fractures. Due to the systemic nature of OP, the increased risk of fractures affects almost all skeletal parts. Hip fractures are specifically dangerous as they are painful and reduce the bearing ability of the patient, which requires surgical fixation and leads to further reduction of functional status and quality of life, decreased mortality, and high medical costs (Compston et al., 2019). OP is a chronic disease that affects men and women of all races, significantly impacting their quality of life and even mortality. Currently, the primary treatment drugs include anti-resorptive agents (such as calcitonin, estrogen, estrogen-receptor modulators, bisphosphonates, and vitamin D, etc.) and anabolic agents (such as teriparatide, strontium ranelate, and romosozumab, etc.). However, although these drugs have therapeutic benefits, their long-term use can cause adverse effects (such as nausea, vomiting, headache, insomnia, anxiety, etc.), which has prompted researchers to search for alternate effective and safe OP therapies (Ukon et al., 2019; Martiniakova et al., 2020).

The application of stem cell technology in clinical practice has opened the path for future medical development (Zakrzewski et al., 2019). Mesenchymal stem cells (MSCs) are pluripotent stem cells found in the mesoderm, which have strong proliferation ability and multi-directional differentiation potential. MSCs have been observed to continuously migrate from their original tissue to new tissue sites, where they participate in the renewal and repair of tissues and organs under physiological or pathological conditions, to maintain the integrity and functional stability of the body’s tissue morphology. With continuous technological progress and advanced medical methods, MSCs-based cell therapy has been increasingly employed to treat various clinically refractory diseases, becoming one of the current research hotspots and the most mature type of cells in clinical research and application (Yamanaka, 2020). Bone mesenchymal stem cells (BMSCs) are important members of the stem cell family, which can differentiate into various cell types such as OBs, adipocytes, chondrocytes, and fibroblasts. Furthermore, BMSCs are crucially involved in the regulation of bone homeostasis. A study on degenerative bone and joint diseases in the elderly, such as OP, indicated that MSCs have good bone and cartilage repair capabilities, highlighting a novel target for the treatment of such diseases (Jiang et al., 2021). Traditional Chinese medicine (TCM) has been employed for centuries for the prevention and treatment of OP. Several studies have indicated that TCM and its formulas can exert therapeutic effects on OP by inducing the proliferation and osteogenic differentiation of BMSCs. This review summarizes how TCM herbs stimulate osteogenic differentiation of BMSCs and discusses their application in treating OP.

## 2 BMSCs regulation of physiological and pathophysiological processes in osteoporosis

The OP is a systemic disease characterized by low bone mass and destruction of bone microstructure, resulting in increased bone fragility and fracture susceptibility (Lane, 2006). The bone formation and resorption is a dynamic equilibrium process and OP is usually caused by the imbalance in this equilibrium. This dynamic equilibrium is achieved by the mutual regulation of OB and osteoclasts (OC). The OBs secrete osteoid into the absorption cavity and are responsible for producing and processing new bone materials, whereas OC promotes bone resorption through acidification and proteolytic digestion (Manolagas, 2000). When bone resorption exceeds bone formation, it leads to bone loss and ultimately OP. Pain is the most common symptom of primary OP, with lower back pain being the most frequent. The front of the vertebral body carries a large amount of weight, therefore, it is prone to compression and deformation, which causes the spine to tilt forward, forming a hunchback, and even fractures. Fractures are the most common and severe complication of degenerative OP (Aibar-Almazán et al., 2022). It has been indicated that age-related bone loss is the predominant cause of OP. In children and adolescents, bone formation exceeds bone absorption, and bones grow in size, and strength, and have increased mineral content. During middle age, in women after 55 years (especially after menopause) and in men after 65 years, the OB’s function gradually decreases, and the OC’s bone absorption function increases, leading to enhanced bone absorption and reduced bone formation (Compston et al., 2019). Therefore, as the age increases, the mineral, organic matrix, and bone mass tend to decrease, reducing the mechanical strength of bone and making it prone to OP. In women, OP is often associated with increased cancellous and cortical bone remodeling, coupled with weight-bearing plastic balance, leading to bone loss and bone microstructure destruction. In men, OP is mainly related to reduced bone formation, low bone turnover, changes in the matrix and mineral composition, etc. (Liu et al., 2015). In addition to age, genetic, endocrine, and nutritional factors, as well as lifestyle, living environment, oxidative stress, and inflammatory response are also considered the risk factors of OP. For example, in white and Asian populations, the incidence rate of OP in elderly women is higher, while in Hispanics, the incidence rate of OP in men is higher than that of women; however, after the age of 60, women have higher OP incidence than men (Bohinc and Snyder, 2008). The cessation of ovarian function and estrogen deficiency is the main cause of OP in postmenopausal women, and there may also be a relative increase in parathyroid hormones (Li and Wang, 2018). Calcium is the most important bone mineral, and its insufficiency inevitably affects bone mineralization. During the bone growth and development period, the calcium requirement increases and insufficient intake can induce OP (Weaver et al., 2016). In addition, smoking, excessive drinking, a high salt diet, excessive coffee consumption, and reduced light exposure are all risk factors for OP (Hannan et al., 2000).

BMSCs are pluripotent stem cells with the ability to differentiate into various cell types and play a crucial role in the physiological and pathological processes of OP. Under normal physiological conditions, BMSCs promote bone formation and regeneration by differentiating into OB after being stimulated by appropriate growth factors and cytokines (such as bone morphogenetic proteins BMPs). These differentiated OBs then synthesize and secrete bone matrix, and mineralize to form new bone (Lee et al., 2018). However, in OP patients, the BMSC’s osteogenic differentiation ability is significantly reduced, thereby decreasing bone formation rate, which might be associated with aging, hormone level changes, chronic inflammatory status, and other metabolic disorders. The result is a decrease in bone density, bone fragility, and an increased risk of fractures (Zhang et al., 2022). In recent years, several studies on the correlation between BMSCs and OP have indicated that osteogenesis induction BMSCs is an important pathway for bone tissue renewal. As the age increases, the number of BMSCs decreases, and their ability to induce also osteogenesis decreases, thereby reducing the OB proliferation and differentiation as well as bone formation ability, thus increasing the risk of OP incidence (Arthur and Gronthos, 2020; Jiang et al., 2021).

In addition, young BMSCs have high proliferation and differentiation potential, therefore, they can effectively participate in bone formation and repair. However, with increasing age, the proliferation and differentiation ability of BMSCs gradually decreases, and BMSC’s senescence is one of the important factors causing a decrease in bone mass and the destruction of microarchitecture, specifically in senile OP, regarded as a frequent aging-related disease (Boz and Ozsarı, 2020). With age, not only does the number of BMSCs decrease, but their differentiation ability is also reduced. BMSCs can also differentiate into adipocytes. Under physiological conditions, the “osteogenic-adipogenesis” differentiation of BMSCs is imbalanced, where osteogenic differentiation is reduced and adipose differentiation is enhanced (Ivanovska et al., 2015). The accumulation of adipocytes in the bone marrow not only occupies the position of OB but also inhibits their function by secreting adipokines such as leptin and adiponectin, further exacerbating OP. Furthermore, the aging cells secrete senescence-related secretory phenotype (SASP) factors, which further inhibit osteogenic differentiation and promote inflammatory response through autocrine and paracrine pathways, thereby exacerbating OP (Chen et al., 2016). During bone formation, BMSCs are regulated by various cytokines and signaling pathways. The Wnt/β-catenin, Notch, and mitogen-activated protein kinases (MAPK) signaling pathways play important roles in the osteogenic differentiation of BMSCs. These signaling pathways maintain the dynamic balance of bone formation and resorption by regulating the proliferation, differentiation, and survival of BMSCs (Guo et al., 2021; Yang et al., 2023). The above data summarized how the molecular mechanisms modulate BMSC function associated with OP, highlighting that BMSC can be used as a therapeutic target for OP treatment.

## 3 The application of Chinese medicinal herbs stimulating BMSCs in osteoporosis

Currently, OP is being treated with drugs or hormonal therapy, which directly stimulates bone formation to increase bone mass. However, prolonged drug treatments have severe side effects. Considering that chronic OP requires long-term and sustained intervention, stem cell regenerative medicine technology is a better treatment alternative (Arjmand et al., 2020). For regenerative medicine, BMSCs are potential candidates as they have fewer ethical concerns than other stem cells investigated for OP treatment. It has been observed that MSCs can treat OP by promoting osteogenic differentiation, increasing the number of OB, inhibiting OC differentiation, and improving bone metabolism, etc. (Hu et al., 2018). TCM has been recommended for bone regeneration and repair for thousands of years. Recently, comprehensive research on TCM and BMSCs has indicated that the TCM has a unique advantage of stimulating BMSCs for the prevention and treatment of OP, demonstrating a wide range of application prospects.

### 3.1 Single herbs and their effective components for osteoporosis treatment

#### 3.1.1 Epimedii folium

*Epimedii folium* (Yinyanhuo in Chinese) [*Epimedium brevicornu Maxim*] is among the most commonly used herbs for OP treatment. Its main active component, such as icariin, has various biological functions, such as the stimulation of bone formation and sexual function, protection of the nervous system, and prevention of degenerative diseases (Chen et al., 2015) (Table 1). Furthermore, the total flavonoids of Herbal *Epimedii folium* (0.006–6 μg/mL) could regulate run-related transcription factor 2 (Runx2)-mediated osteogenesis and peroxisome proliferator-activated receptor gamma (PPARγ)-mediated adipogenesis in BMSCs, further exhibit beneficial effects to bone health (Zhang et al., 2016). Moreover, they (10 μg/mL) have also been observed to regulate the balance between BMSC’s osteogenic and adipogenic differentiation in ovariectomized rats by down-regulating expression of Dickkopf-related protein 1 (DKK1) (Xu et al., 2011). Naturally isolated icariin has gained significant attention as an alternative component for OP treatment, which stimulates bone formation, inhibits bone resorption, and increases angiogenesis (Zhang et al., 2014; Wang et al., 2018). Icariin (10^−5^ M) can induce osteogenic differentiation of pre-osteoblastic MC3T3-E1 cells and mouse primary OB by upregulating the mRNA expression of Runx2, DNA binding inhibitor 1 (ID-1), and bone morphogenetic protein (BMP-4) (Zhao et al., 2008). Scholars have studied the dose-effect relationship of icariin and found that it has a dose-dependent effect (10^−9^ to 10^−6^ M) on the proliferation and osteogenic differentiation of human BMSCs within the low concentration range. Therefore, icariin is extremely low cost and its high abundance has made it an attractive treatment alternative for OP (Fan et al., 2011). Furthermore, Icariin (10^−9^ M) also promotes osteogenesis by reversing the decreased proliferation, increased ROS, decreased matrix metalloproteinases (MMPs), reduced osteogenesis, and enhanced adipogenesis of MSCs caused by steroid-associated factors (Sun et al., 2015; Kim et al., 2017). Icariin can also inhibit glycogen synthase kinase-3beta (GSK3β) and PPARγ pathways, stimulate osteogenic differentiation, and inhibit adipogenesis in MSCs (Sheng et al., 2013). Moreover, Epimedium herb has been observed to prevent OP caused by ovariectomy in rats, which further validated that icariin can increase osteogenic differentiation of rat primary BMSCs in a concentration-dependent manner (10^−5^ μM). It can also improve the expression of genes involved in osteogenesis, such as alkaline phosphatase, bone matrix protein (osteocalcin, osteopontin, and bone sialoprotein), and cytokines (TGF-β1 and IGF-I), etc. (Chen et al., 2007). It has been observed that patients with type 2 diabetes mellitus (T2DM) are more prone to OP. Icariin has been indicated to promote BMSC proliferation and osteogenic differentiation in OP patients with T2DM by up-regulating GLI-1 (Xia et al., 2023). Furthermore, another major component of Epimedium folium, maohuoside A (MHA), also promotes osteogenic differentiation of rat BMSCs via BMP, MAPK, and Wnt/β-catenin signaling pathways by enhancing the mRNA expression of BMP-2, BMP-4, Runx2, β-catenin, cyclinD1, ERK1/2, and p38 MAPK, and might be more effective than icariin (Zhang et al., 2010; Yang et al., 2011; Xu et al., 2012). Pharmacokinetic analysis has indicated that icaritin has a short half-life in the blood and only a trace amount reaches the bone tissues. Therefore, to overcome the limitations of poor targeting and metabolism of icaritin *in vivo*, researchers have developed a bone-targeting liposome that can encapsulate icaritin to further promote bone formation and inhibit fat generation in ovariectomy (OVX)-induced OP mice, possibly via the Akt/GSK-3β/β-catenin signaling pathway (Huang et al., 2018). The above studies indicated that *Epimedii folium* and its effective medicinal components exhibit their strong osteogenic activity by enhancing the osteogenic differentiation of BMSCs and regulating oxidative stress, which partially explains their anti-OP effects.

**TABLE 1:** The main effective components in single herb.

Herbs	Compound	IUPAC name	Molecular formula	Molecular weight	Structure
*Epimedii folium*	Icariin	5-hydroxy-2-(4-methoxyphenyl)-8-(3-methylbut-2-enyl)-7-[(2S,3R,4S,5S,6R)-3,4,5-trihydroxy-6-(hydroxymethyl)oxan-2-yl]oxy-3-[(2S,3R,4R,5R,6S)-3,4,5-trihydroxy-6-methyloxan-2-yl]oxychromen-4-one	C_33_H_40_O_15_	676.7 g/mol	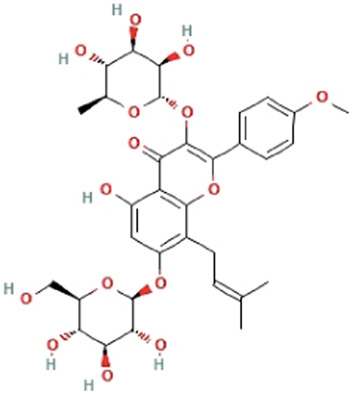
*Ligustri lucidi fructus*	Oleanolic acid	(4aS,6aR,6aS,6bR,8aR,10S,12aR,14bS)-10-hydroxy-2,2,6a,6b,9,9,12a-heptamethyl-1,3,4,5,6,6a,7,8,8a,10,11,12,13,14b-tetradecahydropicene-4a-carboxylic acid	C_30_H_48_O_3_	456.7 g/mol	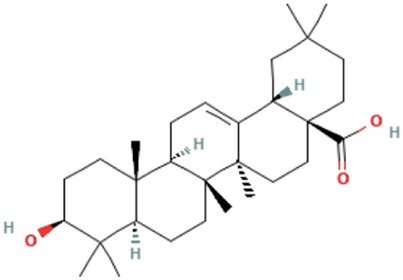
	Oleanolic acid methyl ester	methyl (4aS,6aR,6aS,6bR,8aR,10S,12aR,14bS)-10-hydroxy-2,2,6a,6b,9,9,12a-heptamethyl-1,3,4,5,6,6a,7,8,8a,10,11,12,13,14b-tetradecahydropicene-4a-carboxylate	C_31_H_50_O_3_	470.7 g/mol	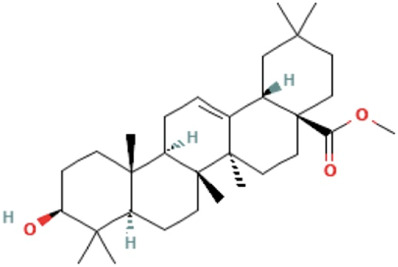
	Ligustroside	methyl (4S,5E,6S)-5-ethylidene-4-[2-[2-(4-hydroxyphenyl)ethoxy]-2-oxoethyl]-6-[(2S,3R,4S,5S,6R)-3,4,5-trihydroxy-6-(hydroxymethyl)oxan-2-yl]oxy-4H-pyran-3-carboxylate	C_25_H_32_O_12_	524.5 g/mol	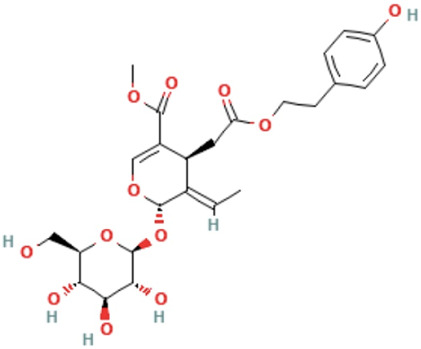
	Ursolic acid	(1S,2R,4aS,6aR,6aS,6bR,8aR,10S,12aR,14bS)-10-hydroxy-1,2,6a,6b,9,9,12a-heptamethyl-2,3,4,5,6,6a,7,8,8a,10,11,12,13,14b-tetradecahydro-1H-picene-4a-carboxylic acid	C_30_H_48_O_3_	456.7 g/mol	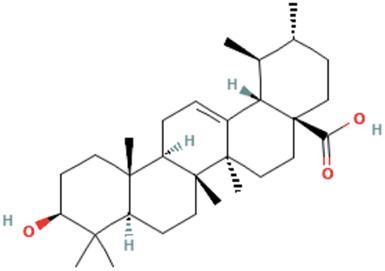
*Psoralere fructus*	Psoralen	furo[3,2-g]chromen-7-one	C_11_H_6_O_3_	186.16 g/mol	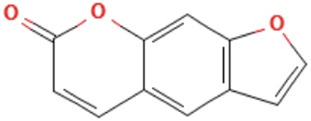
	Angelicin	furo[2,3-h]chromen-2-one	C_11_H_6_O_3_	186.16 g/mol	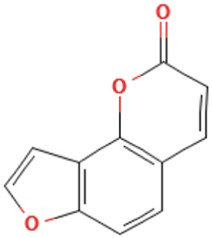
	Bakuchiol	4-[(1E,3S)-3-ethenyl-3,7-dimethylocta-1,6-dienyl]phenol	C_18_H_24_O	256.4 g/mol	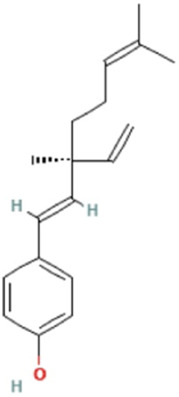
	Apigenin	5,7-dihydroxy-2-(4-hydroxyphenyl)chromen-4-one	C_15_H_10_O_5_	270.24 g/mol	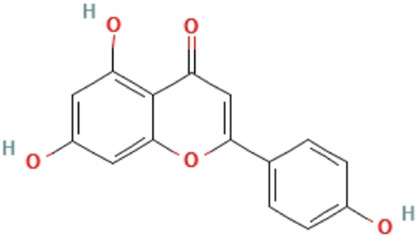
	Kaempferol	3,5,7-trihydroxy-2-(4-hydroxyphenyl)chromen-4-one	C_15_H_10_O_6_	286.24 g/mol	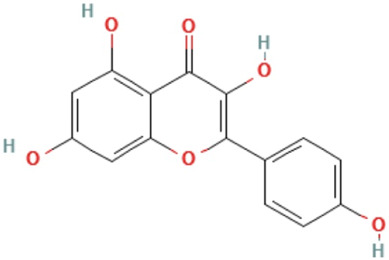
*Drynariae rhizome*	Naringin	(2S)-7-[(2S,3R,4S,5S,6R)-4,5-dihydroxy-6-(hydroxymethyl)-3-[(2S,3R,4R,5R,6S)-3,4,5-trihydroxy-6-methyloxan-2-yl]oxyoxan-2-yl]oxy-5-hydroxy-2-(4-hydroxyphenyl)-2,3-dihydrochromen-4-one	C_27_H_32_O_14_	580.5 g/mol	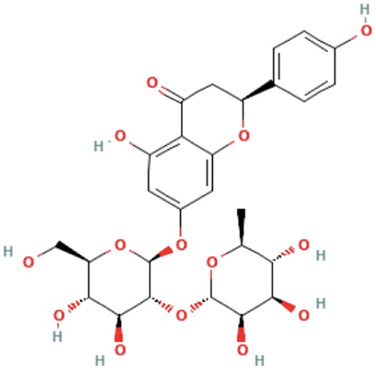
*Morindae officinalis radix*	Nystose	(2R,3R,4S,5S,6R)-2-[(2S,3S,4S,5R)-2-[[(2R,3S,4S,5R)-2-[[(2R,3S,4S,5R)-3,4-dihydroxy-2,5-bis(hydroxymethyl)oxolan-2-yl]oxymethyl]-3,4-dihydroxy-5-(hydroxymethyl)oxolan-2-yl]oxymethyl]-3,4-dihydroxy-5-(hydroxymethyl)oxolan-2-yl]oxy-6-(hydroxymethyl)oxane-3,4,5-triol	C_24_H_42_O_21_	666.6 g/mol	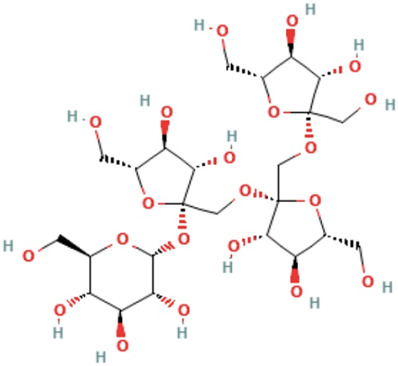
*Angelicae sinensis radix*	Ferulic acid	(E)-3-(4-hydroxy-3-methoxyphenyl)prop-2-enoic acid	C_10_H_10_O_4_	194.18 g/mol	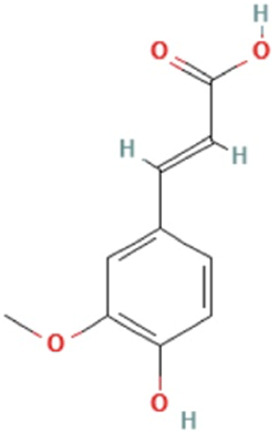
	Imperatorin	9-(3-methylbut-2-enoxy)furo[3,2-g]chromen-7-one	C_16_H_14_O_4_	270.28 g/mol	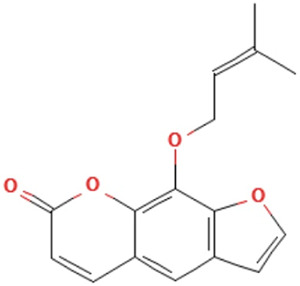

#### 3.1.2 Ligustri lucidi fructus

*Ligustri lucidi fructus* (Nvzhenzi in Chinese) [*Ligustrum lucidum Ait*.] is a commonly used TCM herb for preventing and treating OP by nourishing the liver and kidneys. Its active compounds mainly include ligustroside, oleanolic acid, oleanolic acid methyl ester and ursolic acid, etc. Furthermore, it and its compounds have multiple biological activities including anti-fatigue, anti-oxidation, anti-tumor, and anti-aging properties, etc. (Li et al., 2011). Several comprehensive studies including organic network pharmacology prediction combined with *in vitro* experimental verifications, have confirmed that *Ligustri lucidi fructus* promotes osteogenic differentiation of BMSCs by activating the PI3K/AKT signaling pathway (Kong et al., 2022). *Ligustri lucidi fructus* can significantly increase bone mineral density (BMD) and bone microstructure, whereas reduces body fat content in postmenopausal OP (PMOP) mice. Moreover, it can reduce OC activity, enhance Runx2 inhibit PPARγ, and suppress adipocyte differentiation from BMSCs (Qin et al., 2023). The ethanol extract of *Ligustri lucidi fructus* has been observed to upregulate the expression of osteogenic differentiation regulators (β-Catenin, BMP-2, Cyclin D1, MMPs, Osteoprotegerin, TBX3, etc.), stimulate alkaline phosphatase (ALP) activities, shorten the time required for BMSC’s mineralization during osteogenic differentiation, and is employed for the treatment of OP (Li et al., 2010). The above studies indicated that *Ligustri lucidi fructus* and its effective medicinal components can prevent bone loss and improve bone microstructure by modulating bone and fat balance, which might be a therapeutic agent for OP.

#### 3.1.3 Psoralere fructus

*Psoralere fructus* (Buguzhi in Chinese) [*Psoralea corylifolia L.*] is a herbaceous plant of the leguminosae family, which is commonly consumed for the treatment of OP, bone fracture, osteomalacia, and leukoderma, etc. (Yang et al., 2018). Psoralen, angelicin, bakuchiol, apigenin and kaempferol are the active components of *Psoralere fructus*. It is reported that psoralen can activate the AKT/GSK-3β pathway and increase NRF2 expression to restore BMSCs stemness, thereby further improving the radiation-induced OP (Yin et al., 2022). Moreover, Psoralen can also promote osteogenic differentiation of BMSCs by regulating the expression of osteogenic differentiation-related genes (BMP-4, osteopontin, Runx2, Osterix, and miR-488) and the TGF-β/Smad3 pathway associated proteins (TGF-β1, TGF-β RI, p-Smad, and Smad3) (Huang et al., 2019, 2021). In addition, it has been indicated that Psoralen increases the level of osteogenic marker osteocalcin in ovariectomy (OVX)-induced OP rats, thereby significantly improving bone mass indicators, such as increased trabecular thickness and reduced trabecular space, which may be related to the inhibition of Notch signaling pathway (Yang et al., 2012). Bergapten is a derivative of Psoralen, which belongs to linear furan coumarin and has protective effects against bone loss. The literature suggests that Bergapten can dose-dependently promote ALP, Runx2, and osteocalcin levels in BMSCs *in vitro*. In OVX-induced OP mouse models, Bergapten has been observed to upregulate Runx2 and osteocalcin, and further improve the parameters of bone metabolism such as bone density, trabecular quantity, and trabecular separation, which might be associated with the activation of WNT/β-catenin signaling that can promote BMSCs differentiation into OB (Xiao et al., 2015).

#### 3.1.4 Drynariae rhizome

*Drynariae rhizome* (Gusuibu in Chinese) [*Drynaria fortune (Kunze) J*. *Sm*.] is also a TCM herb, commonly used by orthopedics and OP treatment. Furthermore, it promotes an anti-osteoporotic effect by targeting stem cells, OB, OC, and immune cells as well as multiple signaling pathways (such as PI3K/ATK, OC differentiation, WNT, and estrogen, etc.) (Gan et al., 2019). Moreover, total flavonoids of *Drynariae rhizome* can upregulate the levels of osteogenic-related factors (ALP, OCN, and Runx2), enhance the survival ability, and increase osteogenic differentiation of BMSCs *in vitro*, possibly by activating the ERR1/2-Gga1-TGF-β/MAPK pathway (Han et al., 2024). Naringin is the main effective ingredient of *Drynariae rhizome* and has been found to promote the proliferation and osteogenic differentiation of human BMSCs, thereby providing a basis for the therapeutic mechanism of *Drynariae rhizome* against OP and bone nonunion (Zhang et al., 2009). In addition, naringin dose-dependently increased ALP activity, osteogenic genes mRNA levels (ALP, BSP, and Cbfa1), and Notch1, whereas it decreases the mRNA levels of PPARγ2, suggesting that the osteogenic effect of naringin might be associated with the Notch signaling pathway (Yu et al., 2016).

#### 3.1.5 Morindae officinalis radix

*Morindae officinalis radix* (Bajitian in Chinese) [*Morinda officinalis* How] is a TCM herb that has been used for centuries as tonics for nourishing the kidney, strengthening the bone, and enhancing the immune function in OP treatment (Zhang et al., 2018). It contains potential anti-osteoporotic active compounds such as nystose that can regulate the proteins implicated in ovarian steroidogenesis-related pathways (Liu et al., 2020). *Morinda officinalis* polysaccharides (MOP) are active components isolated from *Morindae officinalis radix*. They have anti-OP medicinal potential and rat BMSCs pretreated with MOP have been observed to promote osteogenic differentiation (upregulating Runx2 and BMP2) or adipogenic differentiation (downregulating CEBPα and PPARγ). In OVX rats, MOP can activate the PTEN/PI3K/AKT pathway, upregulate BMD, bone-derived alkaline phosphatase (BALP), osteocalcin, and miR-21 (targeting PTEN) levels, as well as partially alleviate OP symptoms (Wu P. et al., 2022). The exosomes are essential for intracellular communication and can carry genetic information (miRNAs, mRNAs, DNA, proteins, and liquids) and act on target cells to exert their effects. Furthermore, they can affect bone metabolism and modulate the OB and OC interaction (Shan et al., 2019). MOP has also been observed to increase mean trabecular thickness and number, mean connectivity density, reduce mean trabecular separation/spacing, and increase cortical bone continuity in femoral tissue in glucocorticoid-induced OP rats. In addition, it also increases Runx2 and RANK, whereas inhibits MMP 9 and cathepsin K levels. In MOP-treated rats, BMSC-Exo can inhibit OC differentiation and proliferation, possibly by upregulating miR-101-3p or inhibiting prostaglandin-endoperoxide synthase 2 (PTGS2) (Wu PY. et al., 2022). The emergence of exosomes provides new insights for mediating intercellular communication and information exchange among BMSCs, OB, OC, and other cells. Therefore, further research is warranted.

#### 3.1.6 Angelicae sinensis radix

In TCM, *Angelicae sinensis radix* (Dangui in Chinese) [*Angelica sinensis* Diels.] herb is consumed for “Qi-invigorating,” i.e., stimulating energy metabolism (Chen et al., 2013). Ferulic acid, imperatorin and others are widely available and inexpensive plant extract isolated from *A*. *sinensis radix*, that has anti-oxidative and anti-apoptotic effects (He et al., 2007). It has been indicated that imperatorin activates Runx2, Col1a1, and osteocalcin in rat BMSCs by promoting p-Ser9 of GSK3β and β-catenin nuclear translocation. Moreover, it also promotes the secretion of the upstream factor p-AKT (Ser473) in the Akt/GSK3β/β-catenin pathway, thereby enhancing osteogenesis and inhibiting OC, alleviating OP symptoms (Yan et al., 2020).

### 3.2 Traditional Chinese medicine prescriptions for the treatment of osteoporosis

For decades, TCM formulas have been used for preventing and treating OP and are significantly advantageous over single herbs. A study prepared Chinese herbal extracts from 9 TCM compounds with anti-fatigue and immune regulatory effects, including *Astragalus*, *Cistanche deserticola*, *Dioscorea polystachya*, *Lycium barbarum*, *Epimedium*, *Cinnamomum cassia*, *Syzygium aromaticum*, *A*. *sinensis*, and *Curculigo orchioides*. It was observed that oral administration of this prescription alleviated alcohol-induced bone metabolism abnormalities in middle-aged and elderly male mice, validating that traditional medicinal plant formulas have therapeutic potential against OP (Qian et al., 2021). Danggui Buxue Tang (DBT) is an herbal mixture comprising *Astragalus membranaceus* and *A*. *sinensis*. In comparison with other herbal extracts, DBT treatment significantly enhances OB with an increase in maximum respiration, reserve capacity, glycolytic capacity, and glycolytic reserve. Moreover, DBT also modulates bioenergy metabolism via cellular Ca^2+^ and ROS signaling transduction, thus improving bone diseases such as OP (Kwan et al., 2021). Studies have also shown that the regulatory effect of TCM on OB and OC is achieved by stimulating BMSCs.

An anti-osteoporotic herbal formula containing *Epimedii folium*, *Ligustri lucidi fructus*, and *Psoraleae fructu*s can enhance bone formation and reduce bone reabsorption of BMSCs-derived osteoblastogenesis. This study also indicated that *Epimedii folium* and *Ligustri lucidi fructus* are the main herbs that promote OB differentiation, and their co-treatment had significant synergistic effects (Siu et al., 2013; Ko et al., 2016). Liu’s Zhenggudan formula comprising *Rehmannia glutinosa*, *Caesalpinia sappan*, *S*. *aromaticum*, *Costustoot*, and *Acacia catechu* has demonstrated good clinical therapeutic efficacy against OP. Furthermore, it has been found to induce osteogenic differentiation of MSCs and treat OP by increasing ALP and osteocalcin (Deng et al., 2022). Moreover, the Qing’e formula was prepared from an ancient Chinese recipe comprising *Cortex eucommiae*, Fructus psoraleae, *Semen juglandis*, and *Allium sativum*. It has been observed to regulate bone metabolism and improve bone mineral density in OP patients. In addition, Qing’e formula-treated serum indicated increased differentiation of BMSCs isolated from the proximal femurs of PMOP mice. Moreover, it can upregulate TGF-β1 mRNA level and ALP activity, as well as enhance the recombinant human bone morphogenetic protein-2 (rhBMP-2)-mediated changes in cell morphology, proliferation, and differentiation (Shuai et al., 2015). The *Herba Epimedii*, *Fructus Ligustri Lucidi*, and *Fructus Psoraleae* formula and its component have indicated no cytotoxic effect on MSCs and can promote cell proliferation by upregulating the expression of Runx2, ALP, and OPN in MSCs (Siu et al., 2017). A TCM formula, Jiawei Yanghe Tang (comprising *Radix Rehmanniae Praeparata*, *Colla Cornus Cervi*, *Papaya*, *Mustard seed*, *Tetrandrine*, *C*. *cassia*, *Spatholobus stem*, *Baked ginger*, *Licorice*, *and Ephedra*) was originated centuries ago in the Qing Dynasty and has been used for treating OP. Its-treated serum and direct drug intervention can activate the bone morphogenetic protein-Drosophila mothers against decapentaplegic (BMP-SMAD) signaling pathway, restore the differentiation potential of BMSCs, promote osteogenic differentiation of BMSCs and inhibit their adipogenic differentiation, thereby improving bone density, microstructure damage, and bone metabolism abnormalities in OVX rats (Luo et al., 2022). Taohong Siwu Decoction (THSWD) is a conventional traditional Chinese prescription that has been observed to promote blood circulation and alleviate blood stasis. Furthermore, THSWD also promotes BMSC’s activity, osteogenic differentiation, and migration in a time- and dose-dependent manner. This might be achieved by upregulating VEGF expression and phosphorylation of FAK (Tyr397) and Src (Tyr418) (Lu et al., 2023). Another TCM formula is Er-Xian decoction which is widely used for the treatment of postmenopausal OP. It has been found to increase bone mass in OVX-induced OP mice, enhance osteocalcin levels, and promote BMSC’s self-renewal and OB differentiation. Moreover, it can rescue several gene expressions (upregulating Col1a1, Cthrcl, Posten, and Igfals) associated with OVX dysregulation (Liu et al., 2016). These data provide the basis for further research and evidence for the application of TCM formulas for OP treatment (Table 2).

**TABLE 2:** Effects and potential mechanisms of TCM prescriptions in OP.

Refs.	Model	Prescription	Related behaviors and tests	Biochemical measurements
Qian et al. (2021)	Alcohol-induced osteopenia	Astragalus, Cistanche deserticola, Dioscorea polystachya, Lycium barbarum, Epimedium, *C. cassia*, *S. aromaticum*, *A. sinensis*, Curculigo orchioides	micro-CT	Bone mineral density↑, relative bone volume↑, bone volume fraction↑, trabecular number↑
Kwan et al. (2021)	Osteoblast culture	Danggui Buxue Tang (Astragalus membranaceus and *A. sinensis*)	Mitochondrial energistics, mitochondrial morphology, Mitochondrial membrane potential	Ca^2+^↑, ROS↑, maximum respiration↑, reserve capacity↑, glycolytic capacity↑, glycolytic reserve↑mitochondrial length↑, cristae area↑, total mitochondrial area↑, mitochondria area per total cell area↑
Siu et al. (2013)	Hindlimb unloading tail-suspended rat model	Epimedii folium, Ligustri lucidi fructus and Psoraleae fructus	Bone mineral density, bone micro-architecture, tail-suspension	Bone loss↓, osteogenesis↑, adipogenesis↓, osteoclastogenesis↓
Shuai et al. (2015)	Postmenopausal osteoporosis mice	Qing’e formula (Cortex eucommiae, Fructus psoraleae, Semen juglandis, Allium sativum)	Morphological observation	Alkaline phosphatase↑, TGF-β1↑, rhBMP-2↑
Luo et al. (2022)	Ovariectomy-induced rats	Jiawei Yanghe Tang (Radix Rehmanniae Praeparata, Colla Cornus Cervi, Papaya, Mustard seed, Tetrandrine, Cinnamomum cassia, Spatholobus stem, Baked ginger, Licorice and Ephedra)	micro-CT	BMP↑, SMAD↑, osteogenic differentiation↑, adipogenesis↓, bone density↑, microstructure damage↓
Liu et al. (2016)	Ovariectomy-induced rats	Er-Xian Tang (Curculigo orchioides Gaertn, Herbaa Epimedii, Radix Morindae Officinalis, Radix Angelicae Sinensis, Cortex Phellodendri, Rhizoma Anemarrhenae)	micro-CT	Osteocalcin↑, osteoblast differentiation↑, Col1a1↑, Cthrcl↑, Posten↑, Igfals↑

## 4 Conclusion and prospects

**FIGURE 1:**
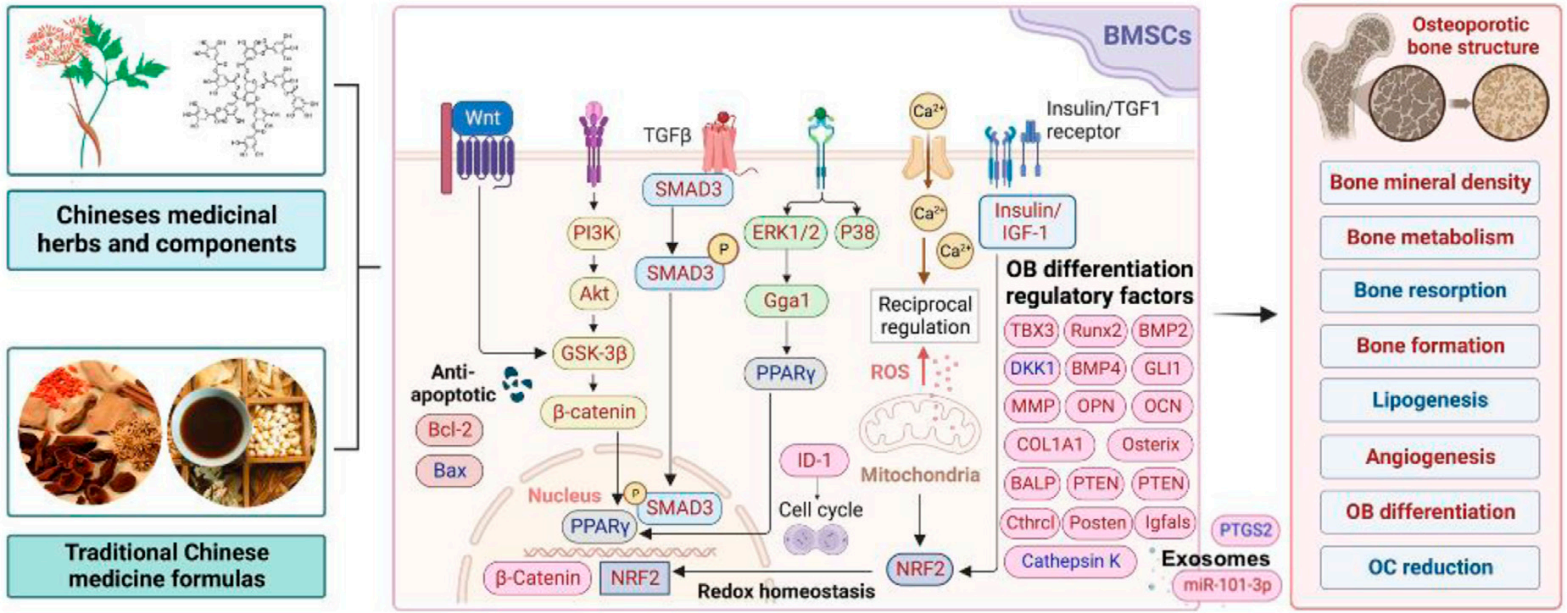
Therapeutic potential of Chinese medicinal herbs stimulating osteogenic differentiation of BMSCs in OP (Note: Factors in red are upregulated by TCM, while factors in blue are downregulated by TCM).

Recent studies have indicated that the BMSC differentiation into osteogenic lineages can promote bone formation and maintain bone homeostasis, which has become a promising strategy for treating OP. Chinese medicinal herbs are safer and more effective substitutes to modern medicine with significant side effects and have been employed for centuries for the treatment of various types of OP. Both *in vivo* and *in vitro* experiments have confirmed that TCM may exert therapeutic effects by stimulating BMSCs differentiation, thereby restoring the balance between OB and OC, inhibiting adipocyte differentiation, and exerting anti-inflammatory, immune regulatory, antioxidant, and other effects (Figure 1). However, clinical research to prove their therapeutic effects is currently insufficient. Some TCM herbs and prescriptions, such as *Dipsaci radix* are commonly used in clinical practice to treat OP and may induce the proliferation and differentiation of BMSCs. This review helps to better understand the mechanism of TCM herbs and their effective components for treating OP, thereby providing a useful and reliable basis for developing more effective anti-OP drugs.

## Abbreviations

OP, osteoporosis; MSCs, Mesenchymal stem cells; BMSCs, Bone mesenchymal stem cells; TCM, Traditional Chinese medicine; OBs, osteoblasts; OCs, osteoclasts; SOP, senile osteoporosis; SASP, senescence related secretory phenotype; MAPK, mitogen-activated protein kinases; DKK1, Dickkopf-related protein 1; Runx2, run related transcription factor 2; PPARγ, peroxisome proliferator-activated receptor gamma; ID-1, DNA binding inhibitor 1; BMP, bone morphogenetic protein; MMPs, matrix metalloproteinases; GSK3β, glycogen synthase kinase-3beta; OCN, osteocalcin; T2DM, Type 2 diabetes mellitus; MHA, maohuoside A; BMD, bone mineral density; PMOP, postmenopausal osteoporosis; ALP, alkaline phosphatase; OVX, ovariectomy; OCN, osteopontin; MOP, Morinda officinalis polysaccharides; MAPK, mitogen-activated protein kinases; BALP, bone-derived alkaline phosphatase; PTGS2, prostaglandin-endoperoxide synthase 2; rhBMP-2, recombinant human bone morphogenetic protein-2.
